# Sympatric Woodland *Myotis* Bats Form Tight-Knit Social Groups with Exclusive Roost Home Ranges

**DOI:** 10.1371/journal.pone.0112225

**Published:** 2014-10-30

**Authors:** Tom A. August, Miles A. Nunn, Amy G. Fensome, Danielle M. Linton, Fiona Mathews

**Affiliations:** 1 Centre for Ecology and Hydrology, Wallingford, Oxfordshire, United Kingdom; 2 University of Exeter, Exeter, Devon, United Kingdom; 3 Biosciences, College of Life and Environmental Sciences, University of Exeter, Exeter, Devon, United Kingdom; 4 Wildlife Conservation Research Unit, Oxford University, Oxford, Oxfordshire, United Kingdom; 5 Biosciences, University of Exeter, Exeter, Devon, United Kingdom; University of Western Ontario, Canada

## Abstract

**Background:**

The structuring of wild animal populations can influence population dynamics, disease spread, and information transfer. Social network analysis potentially offers insights into these processes but is rarely, if ever, used to investigate more than one species in a community. We therefore compared the social, temporal and spatial networks of sympatric *Myotis* bats (*M. nattereri* (Natterer's bats) and *M. daubentonii* (Daubenton's bats)), and asked: (1) are there long-lasting social associations within species? (2) do the ranges occupied by roosting social groups overlap within or between species? (3) are *M. daubentonii* bachelor colonies excluded from roosting in areas used by maternity groups?

**Results:**

Using data on 490 ringed *M. nattereri* and 978 *M. daubentonii* from 379 colonies, we found that both species formed stable social groups encompassing multiple colonies. *M. nattereri* formed 11 mixed-sex social groups with few (4.3%) inter-group associations. Approximately half of all *M. nattereri* were associated with the same individuals when recaptured, with many associations being long-term (>100 days). In contrast, *M. daubentonii* were sexually segregated; only a quarter of pairs were associated at recapture after a few days, and inter-sex associations were not long-lasting. Social groups of *M. nattereri* and female *M. daubentonii* had small roost home ranges (mean 0.2 km^2^ in each case). Intra-specific overlap was low, but inter-specific overlap was high, suggesting territoriality within but not between species. *M*. *daubentonii* bachelor colonies did not appear to be excluded from roosting areas used by females.

**Conclusions:**

Our data suggest marked species- and sex-specific patterns of disease and information transmission are likely between bats of the same genus despite sharing a common habitat. The clear partitioning of the woodland amongst social groups, and their apparent reliance on small patches of habitat for roosting, means that localised woodland management may be more important to bat conservation than previously recognised.

## Introduction

Approximately a third of all mammal species are bats, and the majority of these are long-lived and social for at least part of the year. Colonies, which can be mixed or single-sex, commonly contain tens to hundreds of individuals [1], [2]. Previous studies of the spatial arrangement of social groups have found that whilst some bat species form social groups occupying exclusive roost home ranges [3], [4] others have broadly overlapping roost home ranges [5]. Modern methods of social network analysis (SNA) offer considerable potential for understanding the behaviour of bats, but previous studies have considered only a single species at a single study site [4]–[11]. To our knowledge, there have been no previous studies on sympatric species, either amongst bats or in other orders, making this the first comparison of social networks between sympatric species within a genus (though see [12] for comparisons of populations without SNA). The present study tests predictions about the social structure and spatial arrangement of two sympatric species based on their ecology and the potential for intra and inter-specific competition for roosting sites. The spatial distribution of bat social groups is important for wildlife conservation, whilst characterising the structures of social networks is fundamental to understanding disease transmission and information transfer.

Our study species were *M. daubentonii* and *M. nattereri*, sympatric medium sized insectivorous bats weighing 7–15 g and 6–12 g respectively [13]. *M. daubentonii* typically forages over water but frequently roosts in woodlands, whereas *M. nattereri* is a woodland specialist [13]. Both species roost in tree holes and man-made structures close to their foraging sites and form nursery colonies during the summer composed primarily of pregnant and lactating adult females. Nursery colonies form in May-June and split up once the once young are independent in August-September [13]. In this paper we define a ‘colony’ of bats as an aggregation of individuals at a single location. Both sexes of *M. nattereri* are highly philopatric, returning from hibernation to spend the summer at the site of their birth [14]. In *M. daubentonii,* males are likely to account for most dispersal whilst females are generally philopatric [15]. It has been proposed that summer bachelor colonies of *M. daubentonii* are excluded from areas of high quality foraging habitat by females [16]–[19]. *M. nattereri* also, though less frequently, form bachelor colonies of up to 28 individuals [20]. Like other *Myotis* bats, both species attend swarming sites, typically cave or mine entrances, from late summer to early autumn. These sites are thought to be important for mating, though mating may also occur at summer roosting sites [15] and hibernaculae [18].

Based on evidence that *M. daubentonii* and *M. nattereri* tend to form female dominated social groups, and that *M. daubentonii* males form separate bachelor roosts [15]–[18], we expected the species' social networks to reflect this structure. We set out to test the following:

- Both species will exhibit long lasting intra-sex associations.
- The physical space within the wood occupied by roosting social groups will overlap, since tree dwelling bats change roost site frequently and potential roosts are in excess at this study site.
- *M. daubentonii* bachelor colonies will occupy roosts further from the highest quality foraging sites (in this case, The River Thames and Farmoor Reservoir) due to competitive exclusion by female maternity groups from roost sites closest to foraging areas [16]–[18].

## Materials and Methods

### Fieldwork

Bats were captured and ringed between May and mid-October annually, from 2006 to 2010, at Wytham Woods, Oxfordshire, UK (Latitude: 51.7743, Longitude: −1.3379). This 415 hectare site is composed of semi-natural ancient deciduous woodland and 18^th^–20^th^ century plantations. Over 1150 woodcrete bird boxes of very similar design are dispersed through the woods, many of these are occupied by blue tits (*Parus caeruleus*) and great tits (*Parus major*) until chicks fledge in the second half of May. After this time the boxes are not used by birds but are frequently used by bats up until mid-October, after which the bats migrate to unknown hibernation sites. To minimise disturbance, boxes were not checked more than once within a two week period and females with attached young were not handled. Areas with higher occupancy rates (*pers. obs.*) were sampled more frequently to maximise data collection (Figure S1). Bats were ringed with 2.9 mm aluminium armbands bearing a unique identification number. The individuals were classed juvenile if the joints between the metacarpals and phalanx were not fully ossified [21], [22].

### Ethical considerations

All methods were approved by the University of Exeter Biosciences Ethical Review Committee and by the University of Oxford Ethics Committee and conducted in accordance with Oxford University's Local Ethical Review Procedures, overseen by the Zoology Department Local Ethical Review Committee. The work and was conducted under permit to conduct research within Wytham Woods. Rings were supplied by The Mammal Society, UK and were applied under Natural England licence no. 20113601 and previous licences.

### Social network analysis

A social interaction, or ‘association’, was considered to exist between two individuals if they were found roosting together. Our analyses made no assumptions about the direction of association, as this would require identification of which individuals initiated and which received the behaviour. The link between a pair is therefore scored once in the data we present (ie. the link A-B is not considered separately from B-A). Since the sample size of this study was limited due to the practicality of fieldwork, associations were assumed to be binary and were not given weighting [9].

### Structural analysis

Construction of the association matrix was undertaken in SocProg [23]. Visualisation of the networks was undertaken using Netdraw v.2 [24] - individual bats are represented by nodes and an association between two individuals is represented by a line connecting them. Individuals captured only once (Table 1) were excluded from the analysis, and individuals captured more than once but which had no associations (n = 10) were also removed.

**Table 1:** Frequency distribution of captured bats by species and sex.

Species	Sex	Number of times captured											% caught> once
		**1**	**2**	**3**	**4**	**5**	**6**	**7**	**8**	**9**	**10**	**Total**	
***M. daubentonii***	**M**	430	118	59	14	7	0	1	0	0	0	629	0.32
	**F**	204	55	33	27	13	11	4	0	2	0	349	0.42
	***All***	*634*	*173*	*92*	*41*	*20*	*11*	*5*	*0*	*2*	*0*	*978*	*0.35*
***M nattereri***	**M**	97	43	18	12	9	2	0	1	0	0	182	0.47
	**F**	101	71	42	40	25	14	8	7	4	3	315	0.68
	***All***	*198*	*114*	*60*	*52*	*34*	*16*	*8*	*8*	*4*	*3*	*497*	*0.60*

Individuals were assigned to social groups using the Girvan-Newman method, which is particularly appropriate for populations with a strong social structure such as those studied here [25]. This top-down method, successively removes the association with the highest value of ‘betweenness’. Betweenness is the number of shortest paths, connecting individuals in the network, which contain a given association. Associations with high values of betweenness are those that connect clusters with otherwise low interconnectivity and by removing them the network is broken down into an increasing number of unconnected components. Each time a new component is created the modularity of the network is calculated [26]. Modularity is derived from all associations from the original network and is the difference between the observed fraction of associations that are within components and the fraction expected if associations connected individuals at random. Modularity ranges from 0 to 1, with values over 0.3 regarded as evidence of social structure [26]. The division of individuals to components that produce the highest modularity value is selected as the best representation of social groups.

Evidence of assortment by sex within social networks was examined using join-count in UCINET [27] using 10,000 iterations. This test compares the number of male-male, female-female and intersex associations in the dataset with the number that would be expected by chance. Degree centrality was used to test the hypothesis that females were more central in the networks than males. Degree centrality is the simplest of the centrality measures and is calculated for each individual as the number of connections to other individuals in the network. Since individuals in a network are not independent, the significance of differences in degree centrality was calculated using permutation tests implemented in UCINET [27].

### Spatial Analysis

Roost home ranges of social groups were estimated using 100% minimum convex polygons (MCPs) after the removal of roosts used by single bats (*M. daubentonii*  = 42; *M. nattereri*  = 27) and those isolated by over 1 km (*M. daubentonii*  = 1; *M. nattereri*  = 2) using ArcMap (ESRI v. 10.1, 2010) and Quantum GIS [28]. MCPs were created and cropped so that habitats such as grassland, which do not provide roosting opportunities, were removed.

Radio-tracking was undertaken in August 2009 and 2010 to compare roost home range estimates produced from the SNA to the roost use of individual bats. Three adult female *M. daubentonii,* known to have been present at the site for at least two consecutive summers (one parous but not breeding in the current season, and 2 post-lactation), and one juvenile female were fitted with radio transmitters weighing 0.35 g or 0.42 g (Holohil, Canada, type LB-2N). All tags weighed less than 5% of the body weight of the bat (4.1–4.7%) and were attached by a licensed bat worker using a previously described method [29]. Bats were located at their day roosts using an Australis receiver (Titley Electronics Ltd, Australia) and aYagi 3-element directional antennae (Biotrack Ltd, Wareham, UK). Tree roost locations were recorded by GPS and mapped using ArcMap (ESRI, USA).

### Temporal analysis

The temporal structure of associations was examined using the lagged association rate [10], [30]. This gives the probability that, after being found together, two individuals will be found together at a set time interval in the future. These trends were calculated for each of the four classes of association within each species (male-male, female-female, male-female and female-male) and compared to the expected trend if individuals were to associate randomly – the null association rate. Because individuals are included in the analysis only up until the point where they are last observed, emigration (or mortality) does not influence the values, except in the case of long-term migrations where an individual, having left the population subsequently returns to roost with the same companion. Standard errors were calculated for these trends by jack-knifing the data over a period of 30 days. We note that the error estimates produced using this method have previously been found to be too small, but the method allows for a more reliable interpretation of the data than other available techniques. We therefore recommend that the conclusions should be tested with more data [31].

### Statistical analysis

All statistical analyses were undertaken in R version 2.11.0 [32]. The association between social group's roost home range size and the number of individuals in the social group, species and sampling effort was examined using multiple linear regression. Sampling effort was calculated as the average number of recaptures per individuals for each social group.

## Results

Over 5 consecutive summers we performed 7578 box checks, finding bats on 627 occasions. Bats used numerous different boxes, but at any one time, only a minority were occupied, indicating an excess of potential roosting locations. For example, in 2010, bat droppings were found in 751 of 2279 box checks (33%), but only 146 (3%) had bats present. The two target bat species were never found in the same roost simultaneously, however, 27 roosts (of 293) were used by both species at different times (Figure S2). This is not significantly different from the number of boxes we would expect the species to share if they were randomly selecting empty roosts within the woods (χ2 = 0.48, df = 1, p = 0.49).

A total of 490 *M. nattereri* and 978 *M. daubentonii* were ringed from 379 colonies. Of these, 643 bats were caught more than once, with the mean recapture frequency being 3.6 (range 2–10) for *M. nattereri* (n = 299) and 2.9 for *M. daubentonii* (range 2–9, n = 344)) (Table 1). *M. nattereri* colony size ranged from 2 to 35 individuals (median 7, n = 59), while *M. daubentonii* colonies ranged in size from 2 to 26 (median 10, n = 84). Due to limited opportunities for sampling during the nursery period, and the restrictions this placed on sample size, it was not possible to analyse the social structure separately for each year of the study. All data were therefore combined for SNA, and the nursery and post-nursery periods were considered together. While this approach means we are unable to examine the change in social structure between seasons and years it increases our confidence in results that show social isolation. If 2 social groups are found to have never associated when using 5 years data, we believe this is strong evidence that they do not socialise. The proportion of males captured in the adult population was 0.28 for *M. nattereri* and 0.62 for *M. daubentonii* (Table 1), whereas there was no sex ratio bias for juveniles (0.55 for *M. nattereri* and 0.54 for *M. daubentonii*). Given the relatively low sample size for juveniles (n = 77 *M. nattereri*; n = 98 *M. daubentonii*), we combined data for adults and juveniles in subsequent analyses.

### Social structure of bat populations

For both species, female-female associations were significantly more frequent, and intersex associations significantly less frequent, than would be expected by chance (10,000 iterations, *p*<0.001). Male-male associations were significantly more frequent in *M. daubentonii* (10,000 iterations, *p* = 0.023) and less frequent in *M. nattereri* (10,000 iterations, *p*<0.001) than expected by chance. In both species, females had higher degree centrality than males (one-tailed t-test, 10,000 permutations, *p*<0.001).

**Figure 1:**
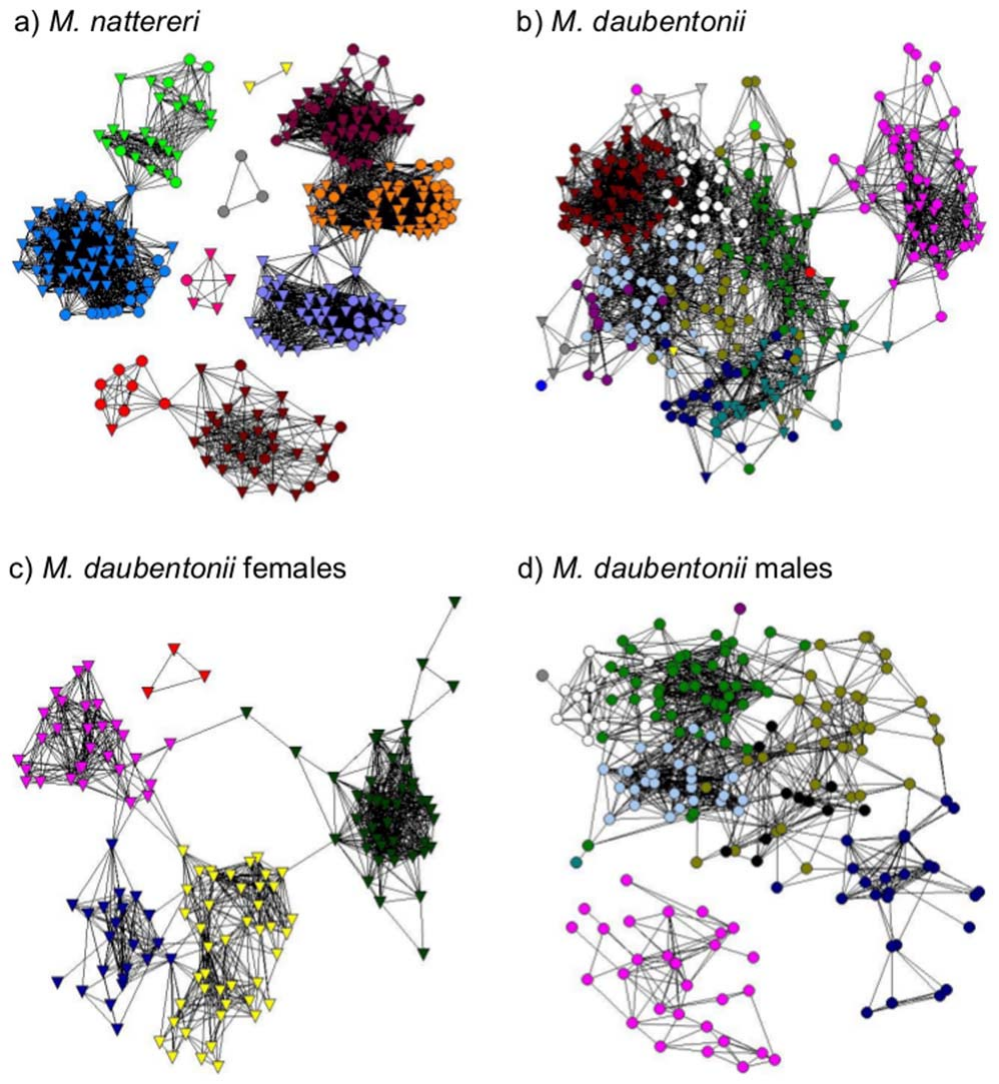
Social network visualisation a) male and female *M. nattereri*, b) male and female *M. daubentonii*, c) female *M. daubentonii*, and d) male *M. daubentonii.* a) *M. nattereri* male (n = 85) and female (n = 214), modularity  = 0.74, b) *M. daubentonii* (n = 344), modularity  = 0.66, c) female *M. daubentonii* (n = 145), modularity  = 0.67, d) male *M. daubentonii* (n = 199), modularity  = 0.64. Nodes represent individual bats (males, circles; females, triangles) and associations are represented by the lines that join them. Colours indicate the assignment of individuals to social groups using the Girvan-Newman algorithm. Colours do not correspond between panels. Colours in a) and c) are comparable to Figure 3. The position of individuals within these networks indicates their position in social space and is not an indication of an individual's geographical location.

*M. nattereri* formed 11 social groups (Figure 1a) with 6 unconnected components. Despite evidence of assortment by sex, *M. nattereri* social groups were composed of a mix of males and females suggestive of a single social group with females at the core. Inter-group associations by either sex were rare, making up only 4.3% of all associations (n = 4258).

*M. daubentonii* also formed discrete social groups, with half of these containing more than 90% males (Figure 1b). This sexual segregation was apparent even when the analysis considered only males recaptured in two or three years, thereby removing ‘transient males’ who may only have been at the study site briefly. Consequently male and female *M. daubentonii* social networks were analysed separately. Individuals in the female network were assigned to 5 social groups (Figure 1c), with inter-group associations making up only 2.1% of all associations (n = 1091) (22 intergroup associations compared to 1069 intragroup associations). Males were assigned to 10 social groups (Figure 1d), however unlike the female *M. daubentonii* networks (and the *M. nattereri* network), there was a significant number of inter-group associations (15.4%, n = 1205, Figure 1d). This interconnectivity suggests that social group membership of *M. daubentonii* males is less specific than for the other networks. This, together with the relatively small sample size, precluded sensible assessment of the spatial organisation of male *M. daubentonii*.

### Spatial distribution of social groups

**Figure 2:**
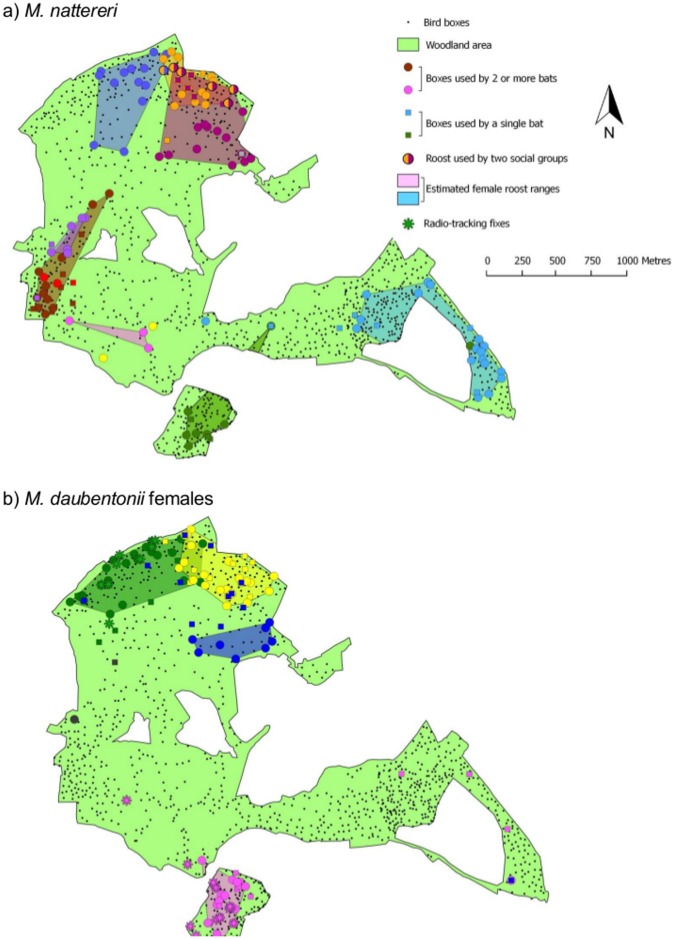
Distribution of a) *M. nattereri* both sexes and b) female *M. daubentonii* social groups in Wytham Woods. Roosts used by bats, and home range estimates are coloured according to social group - colours are comparable to Figure 2, panels a) and c) – symbols indicate colony size and roosts identified by radio-tracking. Roost home ranges are estimated using 100% minimum convex polygons (MCPs). MCPs exclude roosts occupied by a single individual (*M. nattereri*, n = 42; *M. daubentonii*, n = 44) or separated by over 1 km from a roost of the same social group (n = 1 for each species). Four adult female *M. daubentonii* were radio-tracked; two from each of two social groups. The daytime roosts (including trees) used by these individuals are indicated by asterisks and are coloured according to the social group to which they belonged.

Social groups showed roost site fidelity, each restricted to a sub-section of the woodland (Figure 2a and b). The four social groups for which 3 or fewer roosts were known (Figure 2a) were excluded from further consideration as accurate roost home range estimations were not possible. The mean minimum roost home range estimates were 0.16 km^2^ (n = 4, range 0.09–0.30 km^2^) for female *M. daubentonii* and 0.15 km^2^ (n = 7, range 0.02–0.32 km^2^) for *M. nattereri*. There was little spatial overlap between the estimated roost home ranges within species (female *M. daubentonii*  = 5.9%, *M. nattereri*  = 9.4%), and no area was shared by more than 2 social groups (Figure 2a and b). Between species however, there was significant overlap; 32% of the total area covered by both species was shared. Roost home range estimates were positively correlated to the number of individuals assigned to a social group (F = 6.62, df = 1, P = 0.03) but were not linked with sampling effort (F = 0.05, df = 1, P = 0.82) or species (F = 0.66, df = 1, P = 0.44). The spatial distribution of social groups did not reflect our sampling regime, and areas surveyed in a single day frequently contained more than one social group (an example of daily sampling is shown in Figure S1).

The roosting sites of four female *M. daubentonii*, two from each of two different social groups, were identified using daytime positioning for 51 tag-days (10–15 days per bat). These individuals were located in boxes on 29% of tag-days (range, 20–55%), and in natural tree roosts on all other occasions. The tracked bats changed roosts on average every 2 days (range, 1.1–3.5) and were located inside the roost home range of their group on 75% of occasions (range, 30–71%; Figure 2b). Of those roost locations that were outside the minimum roost home range, 28% were within 15 m and 42% were within 100 m of their range. On no occasion was a radio-tracked individual located in the known roost home range of another *M. daubentonii* social group, despite several other potential ranges lying within easy flight distance. *M. daubentonii* bachelor colonies observed during the nursery period were frequently found within the estimated roost home ranges of female social groups and of these, 5 bachelor colonies were identified in roost locations previously used by nursery colonies (Figure S3).

### Duration of association between individuals

**Figure 3:**
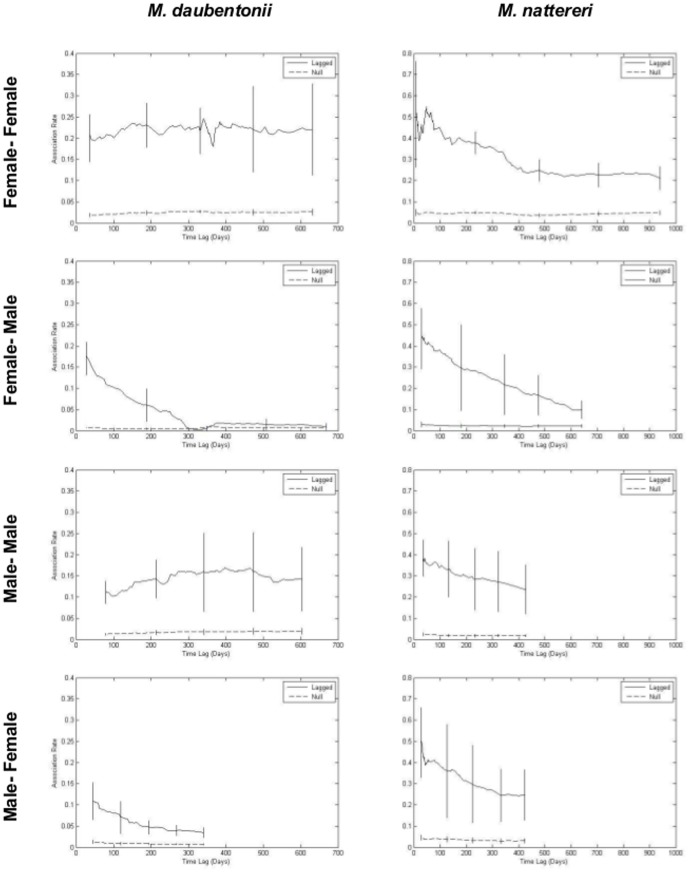
Lagged association rates within and between sexes of *M. daubentonii* (left) and *M. nattereri* (right). Standard error is calculated by jackknifing over a 30-day period.

Associations within and between sexes differed in their stability over time. This was true for both species (Figure 3). Up until approximately 400 days, all classes of *M. nattereri* association showed similar lagged association rates (Figure 3). Approximately half of associating pairs of *M. nattereri* were found associating after a few days, dropping by 100 days to 35–45% (range encompasses means, by sex, of the probability of a bat being found in future with the same individual i.e. M-M, M-F, F-M and F-F). There was then a gradual decline across all classes of association until about 400–500 days, suggestive of the breakdown of casual acquaintances. Beyond this point data were lacking for male-male and male-female associations; however female-male associations showed a continued decline suggestive of further breakdown of casual acquaintances, while female-female associations stabilised. These results suggest that *M. nattereri* have casual relationships lasting up to 400 days with some constant companionship between females beyond this. Overall there was some suggestion that the lagged association rates of same-sex associations were higher than those between sexes (Figure 3). Lagged association rates for all classes stayed above the null association rate for all time intervals, indicating the presence of preferred associations both within and between sexes.

Different trends are observed in the temporal structure of *M. daubentonii* associations (Figure 3): only a quarter of the associating pairs were found associating after a few days. After this point, same-sex associations approximated those seen for female-female *M. nattereri* after 500 days, indicating that stable long-term companionships exist. By contrast, between-sex associations showed a decline in lagged association rate following the first few days, plateauing at 100 days for male-female associations and 300 days for female-male associations (Figure 3). After these time points there was little difference between the observed level of association and that expected from a random network (null association rate) suggesting that between-sex associations amongst *M. daubentonii* represent casual acquaintances that last no longer than a year. Association rates for both species are below 50%, even at short time periods, indicating that most bats do not roost with the same individuals every day.

## Discussion

This study identified multiple social groups in both *M. nattereri* and *M. daubentonii* populations within a continuous landscape in which roosts are not limiting. The social groups formed by *M. daubentonii* females and *M. nattereri* of both sexes show few inter-group interactions and little overlap between roost home ranges (Figure 1a, c and Figure 2a, b).

### Social structure

As expected from work on other species [11], both *M. nattereri* and *M. daubentonii* formed multiple social groups centred on females. Almost all male *M. nattereri* also associated with only one social group, and male-male associations were less common than expected by chance.

Analysis of the temporal structure of associations support our prediction that intra-sex associations would be long lived. For both *M. nattereri* and *M. daubentonii* we observed enduring female to female and male to male associations. In each case, we found evidence of associations lasting more than one year, meaning that the individuals reformed their associations after prolonged absence from summer roosts during the hibernation period, as has previously been reported in *M. bechsteinii* [33]. Inter-sex associations amongst *M. daubentonii* were found to be short lived, but amongst *M. nattereri*, associations between sexes were seen to last more than a year. In both cases, this difference is likely to be the result of the dispersal behaviour of males, since male *M. nattereri* are thought to be philopatric [14] while a proportion of male *M. daubentonii* are thought to disperse [15].

### Spatial structure

Wytham Woods contains an excess of roost sites, most of which are empty on any given day. We therefore predicted that intra-specific social group home ranges would overlap since competition for roosts is likely to be low. We found high levels of overlap in the roost home ranges of different species, but very little overlap in the roost home ranges of social groups within a species. One potential hypothesis to explain the lack of intra-specific overlap is that social groups are defending foraging resources. *M. nattereri* forage within woodland and it is possible that they are defending patches of woodland from other *M. nattereri* groups whilst *M. daubentonii*, which preferentially forage over water, might defend areas of woodland that give them easiest access to their prime foraging habitats. This could be tested by observing the foraging of individuals from known social groups using radio-tracking.

Previous studies suggest that *M. daubentonii* bachelor colonies are excluded from roosting in areas of high quality foraging habitat by females [15]. We therefore predicted that areas of the study site close to high quality foraging sites (The River Thames and Farmoor Reservoir) would host female colonies but not bachelor colonies. However, while female social groups were indeed found close to these foraging habitats, males were frequently found within the predicted roost home range of female social groups (Figure S3). The domination of territories by females may therefore be dependent on habitat quality, with males being tolerated where resources are abundant as may be the case at our study site. Alternatively there may be sexual segregation of foraging activity outside the woodland.

### Broader implications

The results of this study have implications for bat conservation, disease dynamics, and the transfer of information through the population. Roost home range estimates were very small for both species (0.1–0.3 km^2^ for *M. daubentonii* and 0.02–0.3 km^2^ for *M. nattereri*). In addition, roosts identified by radio-tracking were close to, or within, the calculated roost home range. Thus, despite switching roosting locations frequently, woodland bat social groups rely on a network of roosts within a constrained geographical area. Small scale habitat changes, such as the felling of wood for timber, may therefore have a greater impact than previously suspected. Studies of *Chalinolobus tuberculatus*, a threatened New Zealand bat species, have shown that their social groups have similarly restricted roost home ranges [3]. Within the year following tree felling, individuals in the area had smaller roosting home ranges and used fewer roosts than individuals in areas away from felling [34]. A substantial reduction in available roosting habitat within a social group's roost home range may also increase competition between social groups. It is therefore critical that the needs and locations of social groups are considered when undertaking alterations to woodlands with important bat populations.

The social structures we have identified suggest that pathogens are likely to spread rapidly within social groups of *M. nattereri* but slowly between them. For *M. daubentonii*, similar patterns would be expected for females, whilst in males, spread would also be rapid between groups (15.4% of all interactions were between-group). Such heterogeneity in transmission risk is commonly omitted from models of disease epidemiology, but the inadequacy of traditional random-mixing or frequency-dependent based models in describing many diseases is becoming increasingly apparent [35]. Bats are frequently the focus of infectious disease research: a number of recent outbreaks of highly infectious pathogens are thought to have their origins in wild bat populations [36]–[39], and bats are the ancestral host for some viruses that now infect a range of animals including humans [40]. Since males are primarily responsible for connectivity between female groups of *M. daubentonii* we hypothesise that they are likely to have a high probability of infection, and play an important role in disease transmission, as would be predicted by theoretical models [41]. However, this prediction needs to be tested empirically. In a well-characterised population of meerkats (*Suricata suricatta*), roving males had an increased risk of infection with TB (*Mycobacterium bovis*) but the groups visited were not found to be at increased risk of infection [42]. Not only is the social organisation of wild animals important to disease transmission, it is also likely to affect the transfer of information fundamental for individual and population survival. We suggest that identifying the cryptic patterns of social structure and spacing in bats is an important step towards improved management and conservation.

## Supporting Information

Figure S1 **Distribution of sampling effort.** Points show bird boxes (potential bat roosts). Three polygons show examples of the typical area of boxes checked in a day.
(TIF)

Figure S2 **Spatial distribution of roosts.** *M. nattereri* (red) and *M. daubentonii* (blue) and both species (white). Both species have been found in a large number of roosts though occupy few on any given day, suggesting that roosts are not limiting at this site.
(TIF)

Figure S3 **Distribution of** ***M. daubentonii*** **bachelor colonies (defined as >90% male) observed during the nursery period compared to the MCPs of female social groups.**
(TIFF)
