# Supplementary figures and images for: Sympatric Woodland Myotis Bats Form Tight-Knit Social Groups with Exclusive Roost Home Ranges
Source: PLoS One. 2014 Oct 30;9(10):e112225. doi: 10.1371/journal.pone.0112225 (PMC4214762; doi:10.1371/journal.pone.0112225)

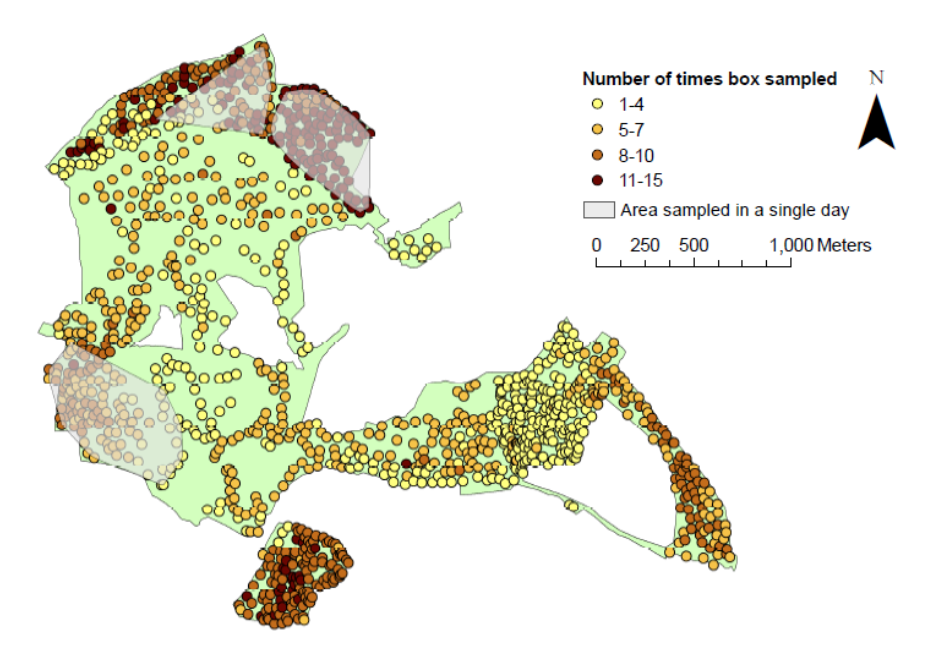

Supplement: Figure S1 — Distribution of sampling effort. Points show bird boxes (potential bat roosts). Three polygons show examples of the typical area of boxes checked in a day. (TIF) [file pone.0112225.s001.tif]

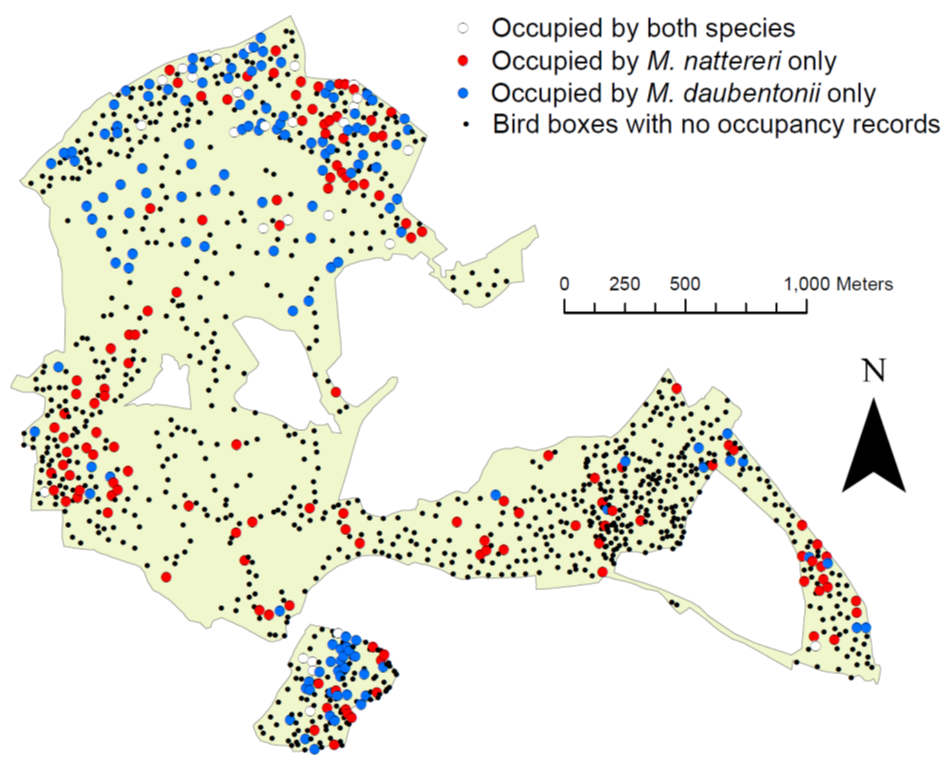

Supplement: Figure S2 — Spatial distribution of roosts. M. nattereri (red) and M. daubentonii (blue) and both species (white). Both species have been found in a large number of roosts though occupy few on any given day, suggesting that roosts are not limiting at this site. (TIF) [file pone.0112225.s002.tif]

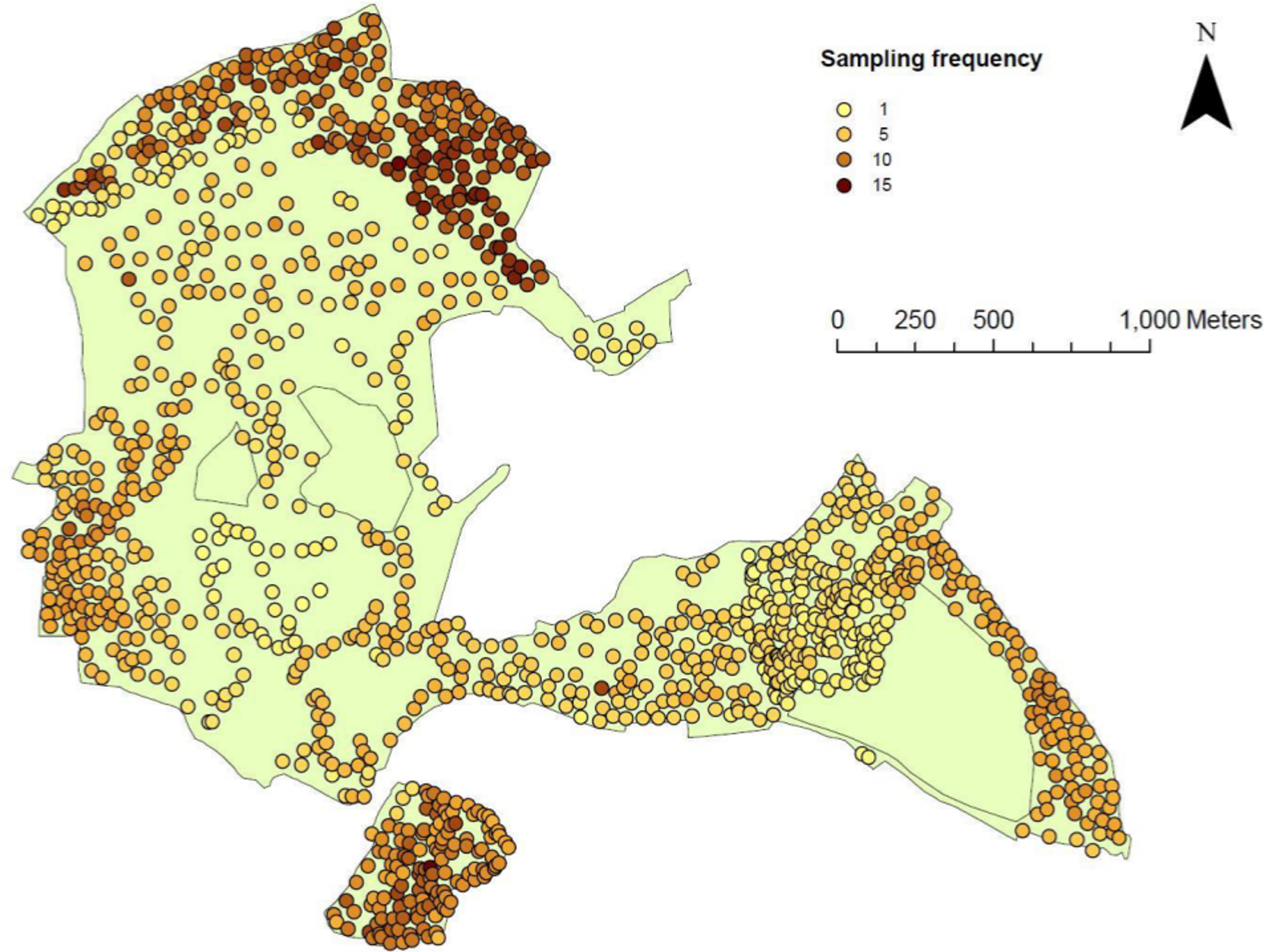

Supplement: Figure S3 — Distribution of M. daubentonii bachelor colonies (defined as >90% male) observed during the nursery period compared to the MCPs of female social groups. (TIFF) [file pone.0112225.s003.tiff]
